# Dual effects of collagenase-3 on melanoma: metastasis promotion and disruption of vasculogenic mimicry

**DOI:** 10.18632/oncotarget.3189

**Published:** 2015-02-26

**Authors:** Xiulan Zhao, Baocun Sun, Yanlei Li, Yanrong Liu, Danfang Zhang, Xudong Wang, Qiang Gu, Jianmin Zhao, Xueyi Dong, Zhiyong Liu, Na Che

**Affiliations:** ^1^ Department of Pathology, General Hospital of Tianjin Medical University, Tianjin, China; ^2^ Department of Pathology, Tianjin Medical University, Tianjin, China; ^3^ Department of Pathology, Tianjin Cancer Hospital, Tianjin Medical University, Tianjin, China

**Keywords:** MMP-13, Melanoma, Vasculogenic mimicry, Laminin, VE-cadherin

## Abstract

Vasculogenic mimicry (VM) is a functional microcirculation formed by tumor cells. Matrix metalloproteinases (MMPs), especially MMP-2 and MMP-9, promote VM formation. Another specific MMP, collagenase-3 (MMP-13), has broad substrate specificity and potentially affects tumor metastasis and invasion. Here we found that MMP-13 was associated with metastasis and poor survival in 79 patients with melanoma. MMP-13 expression was inversely correlated with VM. These results were confirmed in human and mouse melanoma cell lines. We found that MMP-13 cleaves laminin-5 (Ln-5) into small fragments to accelerate tumor metastasis. Degradation of Ln-5 and VE-cadherin by MMP-13 inhibited VM formation. In conclusion, MMP-13 has a dual effect in melanoma, as it promotes invasion and metastasis but disrupts VM formation.

## INTRODUCTION

Melanoma is the least common, but the most serious type of skin cancer [1]. The main cause of death among melanoma patients is widespread metastasis [2]. Essential steps in the metastatic process are the degradation of basement membranes and remodeling of the extracellular matrix (ECM) by proteolytic enzymes such as MMPs which facilities the motility of the tumor cells through ECM [3–5].

Angiogenesis is also crucial during metastasis [6–8]. Three microcirculation patterns can be found in melanoma: endothelium-dependent vessels (EDVs), vasculogenic mimicry (VM), and mosaic vessels lined by both endothelium and tumor cells [9–10]. The VM concept was introduced by Maniotis in 1999 to describe the unique ability of highly aggressive tumor cells to form capillary-like and extracellular matrix-rich tubular networks without the participation of endothelial cells [11], which is reported to be associated with metastasis and short survival in patients with melanoma [12–13].

Various types of MMPs affect melanoma metastasis by degrading ECM [14–15]; MMP-1, MMP-2, and MT1-MMP have essential parts in melanoma VM formation [16].

MMP-13 has broad substrate specificity, and its expression has been found to promote tumor progression and metastasis in a variety of tumors [17–22], including melanoma [23–26]. However, the exact function and possible mechanism of MMP-13 in melanoma metastasis remains unknown. MMP-13 reportedly promotes EDV-dependent angiogenesis in tumors [27–28], and to cause disorganization and fragmentation of tight junction proteins, to enhances the permeability of endothelial cells [29]. As MMP-13 affects endothelium-dependent angiogenesis, its role of MMP-13 in VM formation should be of some interest, but thus far no such data has been available.

This study investigated the role of MMP-13 in the metastasis and VM formation of melanoma using human melanoma tissue samples and cell lines. Specifically, the correlation between MMP-13 and metastasis or VM formation was examined by immunostaining tissue samples. We also performed both an ectopic up-regulation and a short hairpin RNA (shRNA) knockdown of *MMP-13* in melanoma cell lines. Our evidence suggests that MMP-13 is positively correlated metastasis and short survival in melanoma patients, but negatively regulates VM formation in melanoma.

## RESULTS

### MMP-13 is associated with metastasis and poor survival in patients with melanoma

**Figure 1:**
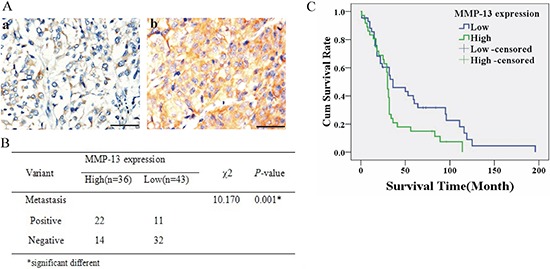
Higher MMP-13 expression (MMP-13^high^) was associated with metastasis and poorer survival of melanoma patients **(A)** Representative IHC-stained images of human melanoma tissues with low (a) or high (b) MMP-13 expression. **(B)** Melanoma with MMP-13^high^ tumor cells has higher metastatic rates than MMP-13^low^ tumors. *P < 0.05. **(C)** Kaplan–Meier survival curve for melanoma patients; the MMP-13^high^ group had a poorer survival rate (*P* = 0.041; bar: 50 μm).

After immunohistochemical (IHC) staining of MMP-13 in melanoma tissues, 36 of 79 tumors were found to have high MMP-13 expression (MMP-13^high^) in tumor cells, whereas 43/79 tumors (including 11 with negative staining) had low MMP-13 expression (MMP-13^low^). Of the 36 patients with the MMP-13^high^ tumors, 61% (22/36) underwent metastasis as compared with 26% (11/43) in the MMP-13^low^ group (Figure 1A, 1B). MMP-13 was located in the cytoplasm of tumor cells, with strongly positive staining at the invasive front of melanoma (Supplementary Figure S1C). Kaplan–Meier survival analysis showed that survival in the MMP-13^high^ group was significantly shorter than in the MMP-13^low^ group (*P* = 0.041; Figure 1C). MMP-13 expression correlated with melanoma thickness and diameter, but not with patient age or sex (Supplementary Table S1).

### MMP-13 promoted invasiveness of melanoma cells *in vitro*

An essential step in metastasis is the degradation and remodeling of ECM by proteolytic enzymes such as MMPs [30]. We used transwell invasion assays to verify the role of MMP-13 in promoting metastasis *in vitro*. The transwell invasion assay is widely used to study motility of different types of cells, including metastatic cancer cells.

**Figure 2:**
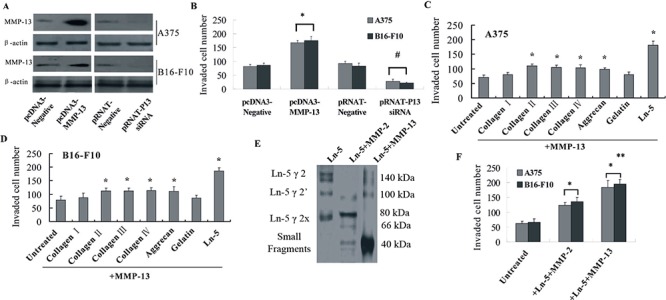
MMP-13 promoted invasion of melanoma cells and cleaved Ln-5 into small molecular weight fragments **(A)** Transfection efficiency was confirmed by western blotting. **(B)** Upregulated or knocked-down MMP-13 expression in A375 or B16-F10 melanoma cells promoted or decreased (respectively) invasion of tumor cells through Matrigel-coated transwell membranes. *P < 0.05, compared with the pcDNA3.1 empty vector control; #*P* < 0.05, compared with pRNAT− control. **(C and D)** Invasiveness of different ECM substrates cleaved by MMP-13. A375 and B16-F10 melanoma cells were incubated for 24 h with products of collagen I, II, III, IV, aggrecan, gelatin or laminin-5 cleaved by MMP-13, and their invasion abilities through Matrigel were measured. Compared with untreated controls, the MMP-13 cleavage products of collagen II, III, IV, aggrecan or laminin-5 all increased invasiveness of the two melanoma cell lines. However, 3× as many cells incubated with the Ln-5, and 2× as many cells incubated with collagen II, III, IV and aggrecan cleavage products invaded the transwell membrane than untreated cells, which suggests the important role of Ln-5–MMP-13 cleavage fragments in melanoma invasiveness. *P < 0.05 compared with untreated control. **(E)** Cleavage fragments of human Ln-5γ2 by MMP-2 or MMP-13 at 37°C for 6 h were run on 12% SDS-PAGE gel and detected by western blot using an anti-human Ln-5γ2-chain antibody. The left lane shows intact Ln-5γ2-chain in 140- and 100-kDa forms. MMP-2 generated an 80-kDa Ln-5γ2x fragment and very faint 66-kDa fragment. MMP-13 further generated low molecular weight fragments of ~40 kDa. **(F)** Adding MMP-13–LN-5 cleavage fragments to the cell culture medium induced melanoma cells to be significantly more invasive than untreated cells and cells treated with MMP-2–Ln-5 fragments. *P < 0.05, compared with untreated controls; **P < 0.05, compared with cells treated with MMP-2–Ln-5 fragments. All of the experiments were repeated three times.

MMP-13 expression levels after transfection were confirmed by western blotting (Figure 2A), which showed that MMP-13 expression was efficiently up-regulated by transfection with pcDNA3-MMP-13 or down-regulated after transfection with pRNAT-MMP-13 siRNA. Corresponding active MMP-13 concentrations in culture media are shown in Supplementary Table S2. We found that melanoma cells with up-regulated MMP-13 expression promoted invasion of tumor cells through Matrigel compared with control cells transfected with empty vector. Knocked-down MMP-13 expression also decreased invading cell numbers (Figure 2B).

Structural ECM changes are necessary for tumor invasion and metastasis. To explore the role of MMP-13 in degrading ECM, MMP-13 digestive products of collagen I, II, III, IV, aggrecan, gelatin or laminin-5 (the main substrates of MMP-13) were added to transwell upper chambers. About twice as many A375 and B16-F10 melanoma cells incubated with MMP-13-digested collagen II, III, IV, aggrecan or laminin-5 invaded the transwell membrane as untreated controls. However, almost *three* times as many cells incubated with cleavage products of Ln-5 invaded the membrane as controls. This implies that Ln-5 degradation is essential to in MMP-13's promotion of invasion (Figure 2C, 2D).

### MMP-13 cleaves Ln-5 γ2 into smaller fragments, which enhanced invasiveness

We examined MMP-13–Ln-5 cleavage fragments to explore their mechanism for increased invasiveness in melanoma, and compared them with MMP-2–Ln-5 γ2 cleavage products, which reportedly also induce tumor cell invasiveness [31]. We found that MMP-2 proteolytically cleaves Ln-5 γ2 into the 105-kDa Ln-5 γ2′ and 80-kDa Ln-5 γ2x. However, MMP-13 could further cleave Ln-5 γ2′ and Ln-5 γ2x into even smaller fragments with molecular weights of approximately 40 kDa (Figure 2E). Our results also showed that adding MMP-13–Ln-5 cleavage fragments to culture medium resulted in more cells invading the Matrigel-coated chambers, compared with both untreated controls and cells treated with MMP-2–Ln-5 cleavage fragments (Figure 2F).

### MMP-13 disrupted VM formation both *in vivo* and *in vitro*

**Figure 3:**
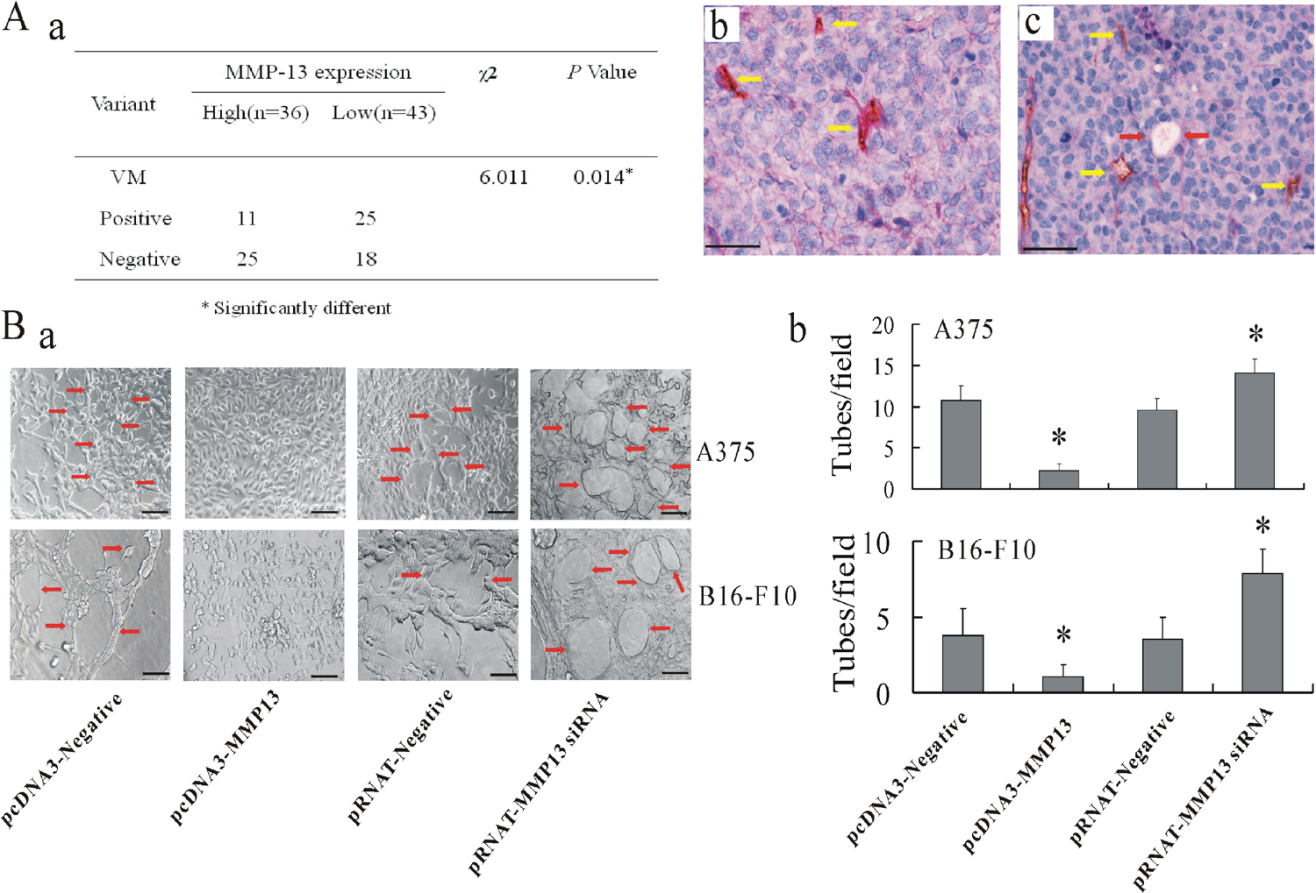
MMP-13 inhibited formation of VM both *in vivo* and *in vitro* **(A)** (a) Significantly less VM can be seen in MMP-13^high^ human melanoma samples than in MMP-13^low^ samples. *P < 0.05. (b) and (c) show representative images of VM in human melanoma tissues with MMP-13^low^ (b) or MMP-13^high^ (c) tumor cells (CD31/PAS double-staining). Yellow arrow in (b) shows endothelial-dependent vessel is CD31^+^ and PAS-positive. *P < 0.05. (c) VM channel formed by tumor cells was CD31^−^whereas base membrane-like structure between red blood cells and tumor cells PAS-positive (red arrow). Yellow arrow: endothelial-dependent vessel is CD31^+^ and PAS-positive. Bar: 50 μm. **(B)** Transfection with pcDNA3-MMP-13 abrogated formation of capillary-like tubes in melanoma cells on Matrigel. However, *MMP-13* knockdown induced typical vessel-like tube formation. (a) Representative images of tube formation in melanoma cells with varying MMP-13 levels. Bar: 100 μm. (b) Quantitative analysis of tubes formed by melanoma cells under different conditions.

MMP-13^high^ melanoma tissues had fewer VM structures, implying an inverse effect by MMP-13 on VM formation (Figure 3A(a, b, c)). Three-dimensional tumor cell culture was used to observe VM formation *in vitro*. Up-regulation of MMP-13 expression in cultured melanoma cells also disrupted formation of capillary-like tubes in Matrigel (Figure 3B(a,b)). By contrast, knocked down MMP-13 expression in melanoma cells induced tumor cells to form more typical ECM-rich vessel-like networks.

### Laminin stained PAS-positive material in the VM loop

**Figure 4:**
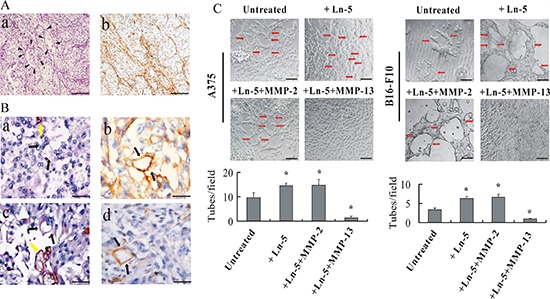
Laminin affects VM formation in human melanoma *in vivo* and MMP-13–Ln-5 cleavage fragments disrupt VM formation *in vitro* **(A)** Dimensional relationship of the PAS-positive patterns and laminin expression in human melanoma. (a) In PAS-stained melanoma tissue sections (without nuclear counterstain), PAS-positive interconnected back-to-back loops and patterns were visible and formed networks (arrow-heads). (b) Anti-laminin immunostaining of human melanoma tissues showed laminin-positive loops and networks similar to the PAS-positive patterns. Bar: 100 μm. **(B)** Dimensional relationship of the VM channels and laminin expression in human melanoma. (a) and (c): CD31/PAS double staining. Black arrows: Vasculogenic mimicry channels in which PAS-positive materials and red blood cells can be seen. Yellow arrow: blood vessels lined by endothelial cells. (b) and (d): Laminin IHC staining. Black arrow: Patterns of laminin-positive cells are not lined by endothelial cells but are in direct contact with melanoma cells. Bar: 50 μm. **(C)** Exogenous addition of MMP-13–Ln-5 cleavage products to 3-D culture medium decreased formation of capillary-like tubes by A375 and B16-F10 melanoma cells, whereas adding Ln-5 or MMP-2–Ln-5 cleavage products formation of *more* typical capillary-like and ECM-rich tubes on Matrigel. Red arrows: vessel-like tubes formed by melanoma cells. *P < 0.05, bar: 100 μm.

Laminin stained the PAS-positive linear structures in melanoma tissues, thereby producing a reticular meshwork pattern (Figure 4A(a, b)). In addition, the PAS-positive material in the VM loop was positively stained with laminin in melanoma tissues (Figure 4B(a, b, c, d)).

### MMP-13–Ln-5 cleavage disrupted VM formation

MMP-2–Ln-5-γ2 cleavage reportedly induces VM formation [16]. We examined whether the different cleavage products of Ln-5 γ2 by MMP-2 and MMP-13 had different functions VM formation. We found that formation of capillary-like tubes by A375 or B16-F10 melanoma cells on Matrigel was almost completely abolished after treatment with Ln-5 cleaved by MMP-13(2 μg/ml), whereas cells treated with exogenous Ln-5 or its MMP-2 cleavage fragments formed more typical vasculogenic-like and ECM-rich networks (Figure 4C).

### MMP-13 cleaves VE-cadherin which may lead to nuclear translocation of β-catenin and transcription of MMP-13

**Figure 5:**
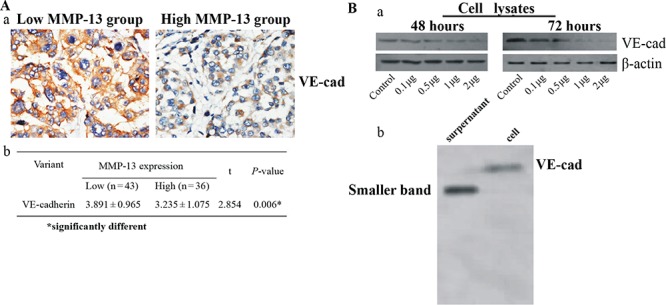
MMP-13 decreases expression of VE-cadherin both *in vivo* and *in vitro* **(A)** (a) Representative IHC stained images of VE-cadherin in human melanoma tissues with low or high MMP-13 expression.VE-cadherin staining can be seen in both membrane and cytoplasm of tumor cells. Bar: 50 μm. (b) Staining index of VE-cadherin in melanoma tissues with high or low MMP-13 expression (mean ± SD). *P < 0.05. **(B)** (a) Different concentration of MMP-13 added to culture medium of A375 for 48 h or 72 h induced dose-dependent decreases in VE-cadherin levels. (b) Western blot analysis of cell lysates and culture medium of A375 stimulated by human recombinant active MMP-13(2 μg/ml) for 72 h, using anti-human-VE-cadherin antibody. Cell lane shows relatively faint band of VE-cadherin at 115 kDa; supernatant lane shows smaller band of VE-cadherin in the culture medium.

VE-cadherin is a marker for VM [32]. Our IHC results showed that VE-cadherin expression was significantly lower in the MMP-13^high^ human melanoma tissues than in the MMP-13^low^ tissues (Figure 5A(a, b)). Also, adding MMP-13 to the culture medium of A375 caused a dose-dependent decrease of the 115-kDa form of VE-cadherin and interestingly, with smaller cleavage fragments in the supernatant medium (Figure 5B(a, b)). Because the B16-F10 melanoma cells express lower levels of VE-cadherin, we used A375 cell lines in this experiment (Supplementary Figure S2).

As MMPs have been shown to interact with a broad range of non-matrix proteins, including adhesion molecules on the cell membrane [33], and MMP-7 reportedly degrades VE-cadherin and induces subsequent nuclear translocation of β-catenin [34], we speculated that MMP-13 might also degrade VE-cadherin. We therefore explored whether degradation of VE-cadherin by MMP-13 could release β-catenin from the VE-cadherin–β-catenin complex.

**Figure 6:**
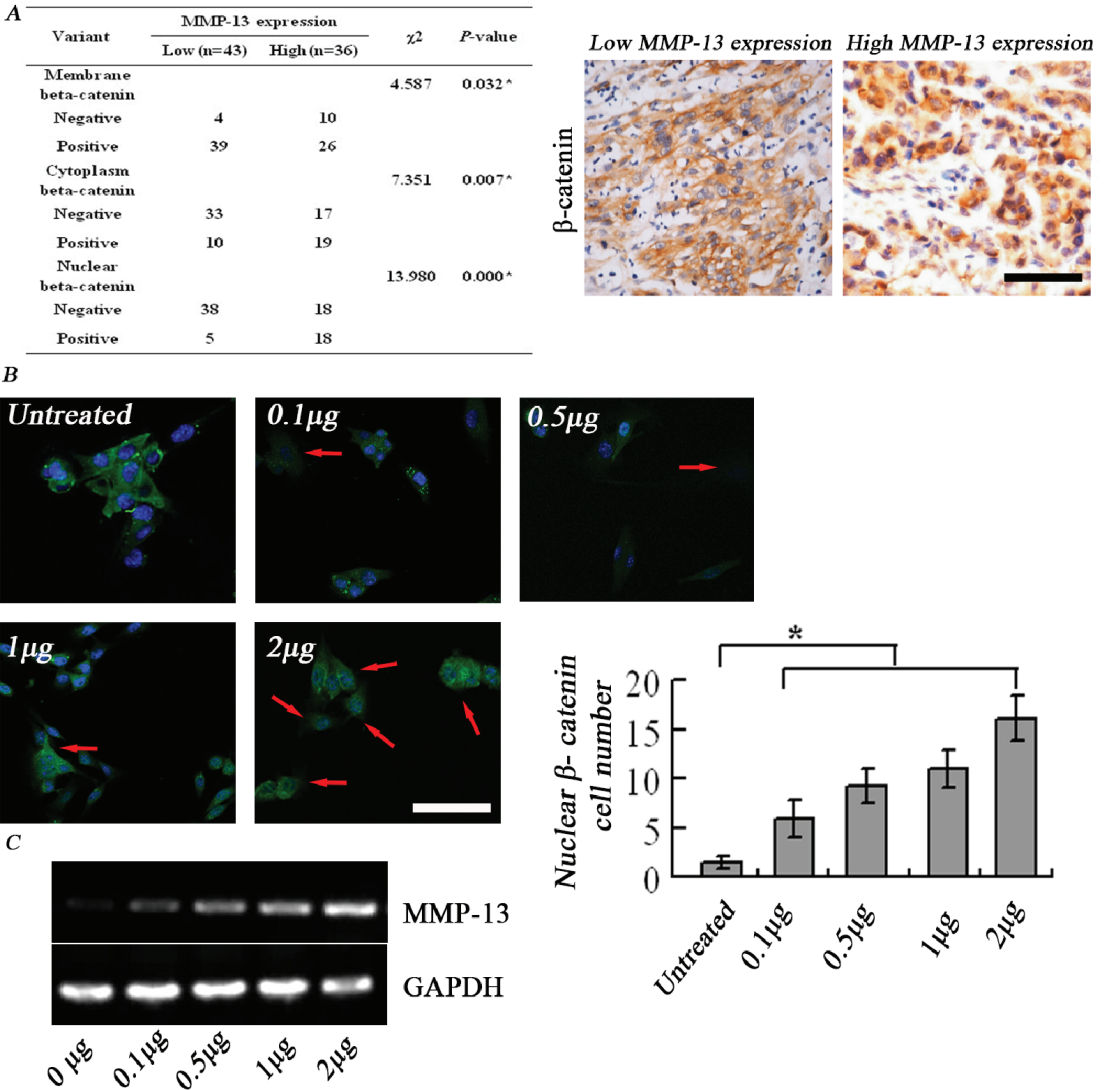
MMP-13 induces nuclear translocation of β-catenin **(A)** Left panel: higher β-catenin^+^ levels in cytoplasm and nucleus are seen in melanoma tissues with high MMP-13 expression than in those with low MMP-13 expression. *P < 0.05, bar: 100 μm. **(B)** β-catenin immunofluorescence staining shows that adding MMP-13 to A375 cell culture medium induces dose-dependent nuclear translocation of β-catenin, from cell membranes in the untreated control group. Number of tumor cells with positive nuclear staining (red arrow) varies with MMP-13 concentration, which is most obvious at 2 μg/ml. *P < 0.05, compared with untreated controls (*n* = 3). Bar: 50 μm. **(C)** After 72 h stimulation with exogenous MMP-13, A375 melanoma cells show dose-dependent increase in *MMP-13* mRNA.

The IHC staining results showed that cytoplasmic and nuclear localization were more easily detected in the high MMP-13^high^ group, whereas membrane-bound β-catenin exhibited an opposite trend (Figure 6A). Exogenous addition of MMP-13 to the culture medium induced nuclear relocation of β-catenin in A375 in a dose-dependent manner (Figure 6B), and induced increased *MMP-13* mRNA levels in A375 cells, which suggests a self-driven loop of MMP-13 production (Figure 6C).

## DISCUSSION

This study demonstrated that MMP-13 has dual effects on melanoma: promotion of metastasis and disruption of formation of VM patterns. From human melanoma tissue samples, we found a positive correlation between MMP-13 expression and metastasis. This result was supported by data that showed MMP-13 to promote invasiveness of cultured melanoma cells, and is consistent with findings that associate MMP-13 with melanoma progression and metastasis [23–26].

However, this study also investigated the mechanism through which MMP-13 promotes invasion and metastasis of melanoma cells. We found that, among tested ECM substrate proteins, MMP-13 cleavage of Ln-5 is had the greatest effect on invasiveness. Our data also showed that MMP-13 further degraded γ2′ and γ2x fragments of Ln-5 (cleavage products of MMP-2 and MMP-14 [35]) into smaller fragments, which is consistent with previous results [31, 36]. We thus speculate that the promotion of invasion and metastasis by MMP-13 occurs through these smaller Ln-5 γ2 fragments, which may facilitate tumor invasion through ECM and promote metastasis.

In this study, we also found that MMP-13 inversely affects VM formation. Reportedly, ECM remodeling by MMP-2 and MT1-MMP promotes VM formation in melanoma by cleaving the Ln-5 γ2-chain into the Ln-5 γ2′ and Ln-5 γ2x pro-migratory fragments, which can be molecular signals in the ECM microenvironment that induce concomitant expression of vascular-associated genes by melanoma cells. [16, 37–39]. This present study showed that Ln-5 fragments cleaved by MMP-13 disrupt VM formation *in vitro*. We thus speculate that MMP-13 may inhibit VM formation by degrading Ln-5γ2′ and Ln-5 γ2x into smaller cleavage fragments that interfere with the molecular signal transition required by the vasculogenic phenotype of tumor cells and ultimately prevent the formation of VM patterns.

A biologically significant marker is necessary to detect VM-associated tumor cells. VE-cadherin is critical in VM formation and often used as a marker for tumor VM [37, 40]. We found MMP-13 may degrade VE-cadherin, in that smaller VE-cadherin cleavage band can be detected in culture medium after MMP-13 treatment. We speculate that MMP-13 inhibits or disrupts VM formation first by cleaving Ln-5 γ2′ and Ln-5 γ2x into smaller fragments, thus preventing transfer of molecular signals in the ECM from inducing the vascular phenotype of aggressive tumor cells; and second by degrading VE-cadherin on tumor cells which may deter the downstream signal transfer and then abrogate the ability to form vasculogenic networks of tumor cells.

Our data also show that MMP-13-caused VE-cadherin degradation on melanoma cells could lead to release of β-catenin into the cytoplasm and nucleus; nuclear β-catenin may thus activate MMP-13 transcription and form a positive feedback loop to amplify β-catenin signaling.

β-Catenin is a subunit of the cadherin adhesion complex and acts as an intracellular signal transducer in the Wnt signaling pathway [41–42]. Several proteases can cleave E-cadherin and VE-cadherin and thus affect β-catenin activity [34, 43]. β-Catenin mainly localizes to the cell membrane. Reduced membranous β-catenin expression combined with accumulation of free cytoplasmic and nuclear pools of β-catenin transcriptionally regulates target genes that affect tumor invasion and/or metastasis [44–45]. We therefore speculate that, apart from degrading Ln-5 into smaller fragments, MMP-13 may promote metastasis by inducing translocalization of nuclear β-catenin.

In conclusion, we have shown that increased MMP-13 expression in melanoma has two effects: promotion of metastasis and disruption of VM formation. These two effects seem contradictory considering that VM is positively correlated with subsequent death from melanoma metastasis [46–47], due to direct exposure of tumor cells to blood flow of blood or their migration along ECM scaffolds [48]. However, we suppose that the process VM disruption by MMP-13 may also facilitate tumor metastasis further. As VM patterns are composed of laminin, which co-localizes with PAS-positive networks [49–50], we propose that MMP-13 degrades the PAS-positive and laminin-positive substances, which constitute the basement membranes of VM; their destruction could thus expose tumor cells more directly to blood flow. Also, MMP-13 may degrade and promote migration along the ECM as well as the physical connection between VM patterns and blood vessels, consequently promoting hematogenous metastasis. Much work would be required to validate this conjecture. Conversely, MMP-13 degrades VE-cadherin, which disrupts VM formation but may also promote metastasis by subsequent nuclear translocation of β-catenin.

Further study will be required to identify the cleaved fragments of Ln-5 γ2. We found two western blot bands of Ln-5γ2-chain at approximately 140 kDa, which were also observed in Seftor's research [16]. The exact reason needs to be elucidated further.

Although much research remains to be done, our results indicate that MMP-13 may be used as a biomarker or therapeutic target in melanoma. Given that MMP13-specific inhibitors have already been developed, this study supports the evaluation of these inhibitors for the treatment of melanoma.

## METHODS

### Patient samples

We obtained 79 primary melanoma specimens and formalin-fixed paraffin-embedded tumor materials from patients who underwent surgical resection in Tianjin Cancer Hospital of China between 5 January 1984 and 20 December 2000, of whom 47 were men and 32 were women. All of the patients died from melanoma-related causes. The diagnoses of these melanoma samples were reviewed by two pathologists.

### IHC staining and assessment

Details of the procedures, assessment methods and the antibodies used are provided in the Supplementary Materials and Methods. Staining index (SI), the sum of staining intensity and the percentage of positive cells, was defined as described previously [51] and used to assess IHC staining. (Representative images of each staining rating and negative control are presented in Supplementary Figure S1A (a, b, c, d), S1B(a, b).) For MMP-13 staining, a SI score >3 was designated as high expression, (MMP-13^high^), whereas a SI score < 3 was designated as low expression (MMP-13^low^). Furthermore, for β-catenin staining, membrane, cytoplasmic, and nuclear staining scores were individually recorded. Staining was recorded as positive when >10% of the tumor cells exhibited positive localization. VM was determined to be PAS-positive channels exclusively lined by tumor cells where red blood cells were present, but not with CD31-stained endothelial cells. The average count of five fields under 400× magnification was recorded as number of VM.

### Cell lines

The A375 human melanoma cell line and the B16-F10 mouse melanoma cell line were obtained from the Cell Resource Center (Beijing, China). The cells were maintained as monolayer cultures in RPMI 1640 medium (Neuronbc Laboratories Co., Ltd., Beijing, China) supplemented with 10% fetal bovine serum (Hyclone, Utah, USA) at 37°C and 5% CO_2_. All the experiments were performed using cultures at 70–80% confluence.

### Expression plasimids and MMP-13 gene silencing

pcDNA3.1 vector and pRNAT vector were used to overexpress and knock down (respectively) MMP-13 in melanoma cells. The vectors were transfected into A375 or B16-F10 melanoma cells by way of percutaneous ethanol injection (Polysciences, Inc., Cat#23966). Stable transfection was selected using G418 at a dose of 600–800 mg/L. The transfection efficiency was confirmed by western blot analysis. The details are provided in the Supplementary Materials and Methods.

### Cleavage of collagen I, II, III, IV, aggrecan, gelatin or laminin-5 by MMP-13

Human purified collagen I, II, III, IV, aggrecan, gelatin or laminin-5(100 ng) (Abcam, UK) was treated with human active recombinant MMP-13 (50 nM) (Millipore, Germany) according to the manufacturer's instruction. The mixtures were incubated for up to 6 h at 37°C in 50 mM Tris (pH 7.5), 150 mM NaCl, 10 mM CaCl_2_ at 37°C before being added to culture medium of melanoma cells in the transwell assays.

### Cleavage of laminin-5 by MMP-2 and MMP-13

Human purified Ln-5 was treated with 50 nM human active recombinant MMP-2 or MMP-13 before being run on SDS-PAGE gels under reducing conditions. These procedures were performed as described previously [35]. The cleavage fragments were detected by western blot analysis as described below, using an anti-human Ln-5γ2-chain antibody (Millipore, Germany).

### Transwell invasion assays, three-dimensional cultures

The details of these procedures are provided in the Supplementary Materials and Methods.

### Immunofluorescence staining, western blot analysis, measurement of active MMP-13 concentration, and PCR

The details of these procedures are provided in the Supplementary Materials and Methods.

### Statistical analysis

SPSS (version 17.0; SPSS, Chicago, IL) was used for all calculations. *P* < 0.05 was considered significant. Spearman's rank correlation test, Student's *t*-test and Pearson χ^2^ analysis were used to evaluate differences between groups. Overall survival was estimated through the Kaplan–Meier method and compared between groups through the log-rank test. Details of statistical analysis method are shown in Supplementary Materials and Methods.

## SUPPLEMENTARY FIGURES AND TABLES


