# Supplementary material for: Dual effects of collagenase-3 on melanoma: metastasis promotion and disruption of vasculogenic mimicry
Source: Oncotarget. 2015 Feb 26;6(11):8890–9. doi: 10.18632/oncotarget.3189 (PMC4496190; doi:10.18632/oncotarget.3189)
Supplement: Supplementary file 1 [file oncotarget-06-8890-s001.pdf]

DETAILED MATERIALS AND METHODS

Immunohistochemistry staning methods

The excised sections were pretreated with citrate buffer (0.01M citric acid; pH 6.0) for 10 min at 95°C in a microwave oven. Endogenous peroxidase activity was blocked with 3% hydrogen peroxide in 50% methanol for 30 minutes at room temperature. Nonspecific binding sites were blocked by exposure to 10% normal goat serum for 20 minutes at room temperature. The sections were incubated overnight at 4°C with primary antibodies (as mentioned above). The staining systems used were PicTure PV6001 and PV6002 (Zhongshan Chemical Co., Beijing, China) and the incubation time was 1 hours at room temperature. The slides were then incubated with 3, 3′-diaminobenzidine (DAB) chromogen for 5~10 minutes and counterstained with hematoxylin. Negative controls were prepared by using PBS in lieu of the primary antibodies and the representative images were shown in Supplementary Figure S1.

The positive staining for MMP-13, VE-cadherin and β-catenin in the melanoma cells was assessed by two pathologists blinded to the patients' clinical pathology parameters using the staining index (SI), the sum of staining intensity and the percentage of positive cells which was defined as described previously[27]. (Representative images of each staining rating are presented in Supplementary Figure S1.). For MMP-13 staining, a SI score ≥3 was designated as "high expression," whereas a SI score <3 was designated as "low expression." Furthermore, for β-catenin staining, membrane, cytoplasmic, and nuclear staining scores were individually recorded. Staining was recorded as "positive" when over 10% of the tumor cells exhibited positive localization.

PAS staining: the slides were exposed to 0.5% periodic acid solution for 15 min and then in Schiff solution for 15~30 min in dark without counterstained with hematoxylin.

For the CD31-PAS double staining, after the CD31 IHC staining was performed, the slides were stained with PAS as described above and then were counterstained with hematoxylin. Normal gastric mucosa was chosen as a positive control.

Expression plasimids and MMP-13 gene silencing

Full-length MMP-13 complementary DNA (cDNA) was generated by normal human or mouse embryo total cDNA, and digested with XhoI/EcoRI and subcloned into pcDNA3.1 vectors. The resulting constructs were confirmed by DNA sequencing. The small interfering RNA (siRNA) kit (pRNAT-MMP-13-shRNA) was purchased from GeneScipt(New Jersey, USA). The target sequence (AACAGCUGCACUUAUCUUCUUAACU) or (GAGCACTACTTGAAATCAT) was used to down-regulate human or mouse MMP-13 *in vitro* respectively. A non-effective shRNA (target sequence 5′-AATTCTCCGAACGTGTCACGT-3′), was used as a negative control.

Measurement of active MMP-13 protein

The concentrations of endogenously active MMP-13 in the cell-conditioned medium were assessed by colorimetry quantification detection kits purchased from Genmed Scientifics Inc (USA) according to the manufacture's guidelines.

Cleavage of laminin-5 by MMP-2 and MMP-13

100 nanogram purified Ln-5(abcam, ab42326) was treated with 50 nM human active recombinant MMP-2 (Cat.No.PF023, Millipore, Germany) or MMP-13 (Cat.No.44287, Millipore, Germany) incubated in 50 mM

Primary antibodies used in this study

Antibody	Company	Country	Clone Number	Species	Pretreatment	Dilutions for IHC
MMP-13	Santa cruz	California, USA	MM0019-12E10	Mouse	Microwave	1:50
VE-Cadherin	abcam	Cambridge, UK	polyclonal	Rabbit	Microwave	1:100
β-catenin	Santa cruz	California, USA	12F7	Mouse	Microwave	1:100
CD31	Zhongshan Biotechnology	Beijing, PR China	JC70A	Mouse	Microwave	Ready to use
Laminin	Zhongshan Biotechnology	Beijing, PR China	polyclonal	Rabbit	Microwave	Ready to use

Tris-HCl (pH 7.5), 10 mM $CaCl_2$, 150 mM NaCl, 1 mM $ZnCl_2$, and 0.02% NaN3 for 6 hours at 37°C. After that, all reactions were stopped with 5× denaturing buffer (0.15 M Tris, pH 6.7, 6% SDS, 20% glycerol, 0.1 M EDTA, 0.03% bromphenol blue). The cleavage fragments were run on SDS-PAGE gels under reducing conditions and detected by western blot as described below using an anti-human Ln-5γ2-chain antibody.

Invasion assays

Invasion ability of melanoma cells were examined using transwell assays. After trypsinization, A375 and B16-F10 melanoma cells (1×10^5) in 100 µl RPMI-1640 without FBS were seeded onto Matrigel coated upper wells with 8 µm porosity polyethylene terephthalate filters (Invitrogen, California, USA). The digestive products of collagen I, II, III, IV, Aggrecan, Gelatin or Laminin-5 were added into the culture medium (2 µg/ml). After 18 h of incubation at 37°C, the upper surface of the membrane was wiped to remove non-invaded cells. The cells that invaded through the Matrigel and adhered to the membrane bottom were stained with crystal violet solution and counted using the method described previously [1]. For the measurement of the effects of MMP-2 or MMP-13 or the invasion ability of melanoma cells, either exogenous MMP-2 or MMP-13 (2 µg/ml) was added into the upper chambers when seeding the cells. The lower inserts used 10% FBS media.

Three-dimensional (3-D) cultures

96-well plates were coated with 20 µl Matrigel which was allowed to gel (1 h, 37°C). A375 and B16-F10 melanoma cells were seeded in complete RPMI-1640 onto the gel and incubated at 37°C for 48 h. Either exogenous Ln-5 or Ln-5 cleaved by MMP-13 or MMP-2 at 37°C for 6 hours was added into the culture medium when seeding the cells (2 µg/ml). Capillary-like structure formation was filmed under phase contrast microscope (200×).

Immunofluorescence Staining

A375 melanoma cells were plated onto chamber slides and cultured in the medium with 10% FBS. Exogenous human recombinant MMP-13 was added into the culture medium at a final concentration of 0.1, 0.5, 1 or 2 µg/ml. The cells were fixed in 4% Paraformaldehyde 72 h after seeding. The primary antibody β-catenin (Clone number: 12F7, Santa Cruz Biotechnology) was diluted at 1:100. The fluorescein isothiocyanate and tetramethyl rhodamine isothiocyanate-conjugated mouse immunoglobulin G antibody (Santa Cruz Biotechnology) were used as labels for immunofluorescence assay. DAPI (Sigma) was used for staining of DNA before been viewed with fluorescent confocal laser scanning microscopy (Nikon, Japan).

Western blot analysis

The whole cell lysates or conditioned medium were resolved by way of SAS-PAGE and transferred onto polyvinylidene difluoride membranes (Millipore). Blots were blocked with 5% free-fat milk for 1 hour at room temperature and then were incubated with the primary antibodies (MMP-13, 1:100; VE-cadherin, 1:500; β-actin) for 1 hour at room temperature, followed by incubation with a horseradish peroxidaseconjugated anti-mouse or anti-rabbit secondary antibody (1:2,000, santa-cruz). Blots were developed using an enhanced chemiluminescence detection kit (ECL; Amersham Pharmacia Biotech, Piscataway, NJ). For protein loading analyses, a monoclonal β-actin antibody (1:2000; Abmart) was used.

Polymerase chain reaction (PCR)

The total RNA was isolated using TRIzol reagent (Invitrogen Life Technologies).

cDNA was synthesized from 2 µg total RNA using Quant reverse transcriptase (Tiangen, China). RT was run for 1 h at 50°C and was stopped by heating for 5 min at 85°C. Reverse transcription (TaKaRa Biotechnology Co., Ltd., Japan) and PCR(polymerase chain reaction analysis, 2×Taq PCR MasterMix, Beijing,China) were used to evaluate the mRNA levels of human MMP-13(35 cycles). Annealing temperature: 65°C for GAPDH; 50°C for MMP-13.

Primer sequences

MMP-13 Forward 5′-TCCCAGGAATTGGTGATA AAGTAGA-3′;

Reverse 5′-CTGGCATGACGCGAACAATA-3′;

GAPDH Forward 5′-CCTGGCCAAGGTCATCCA TGAC-3′

Reverse 5′-TGTCATACCAGGAAATGAGCTTG-3′

Statistical analysis

Relation of MMP-13 expression and VM number was evaluated by Spearman's rank correlation test. Correlation of MMP-13 expression to metastasis, clinicopathologic characteristics or localization of β-catenin were tested via Pearson x2 analysis. Differences of VE-cadherin expression between MMP-13 high and low expression group were detected using a Student's *t*-test.

REFERENCE

1. Seftor EA, Meltzer PS, Kirschmann DA, Pe'er J, Maniotis AJ, Trent JM, Folberg R, Hendrix MJ. Molecular determinants of human uveal melanoma invasion and metastasis. Clin Exp Metastasis. 2002; 19:233–46.

SUPPLEMENTARY FIGURES AND TABLES

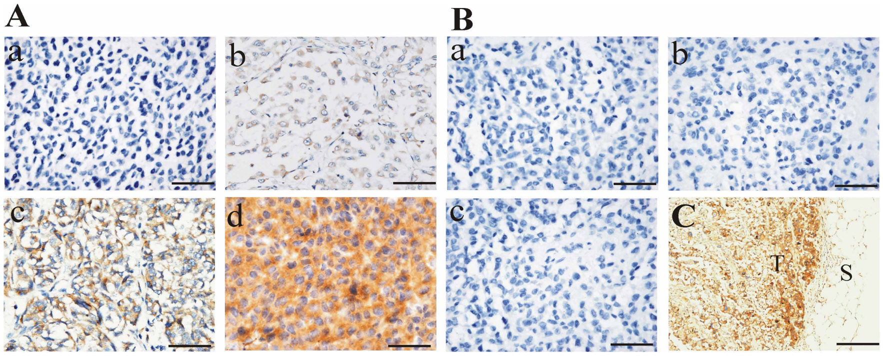

Supplementary Figure S1: Representative images of negative control and staining grades of IHC staining and the representative image of MMP-13 at the leading edge of tumor. **(A)** Representative images of IHC of MMP-13. (a) Negative control of IHC staining of MMP-13. (b) SI score 1 of IHC staining of MMP-13. (c) SI score 2 of IHC staining of MMP-13. (d) SI score 3 of IHC staining of MMP-13. **(B)** Representative images of negative control of IHC staining of VE-Cadherin (a), β-catenin(b) and Laminin (c). **(C)** Representative image of MMP-13 at the leading edge of tumor.

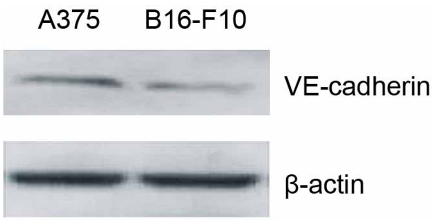

Supplementary Figure S2: Expression levels of VE-cadherin in A375 and B16-F10 melanoma cells.

Supplementary Table S1: Correlation between MMP-13 expression and clinicopathologic characteristics of patients with melanoma

Clinicopathologicalparameters	MMP-13 expression		χ2	*P* Value
	High (*n* = 36)	Low (*n* = 43)		
Age (years)			0.644	0.422
<45	8	13		
≥45	28	30		
Sex			0.072	0.789
Male	22	25		
Female	14	18		
Tumor thickness			33.425	0.000*
≤1mm	3	28		
1.01~2mm	10	9		
2.01~4mm	7	5		
>4mm	16	1		
Tumor long diameter (cm)			8.722	0.003*
<4	15	32		
≥4	21	11		

*significantly different

Supplementary Table S2: Concentration of active MMP-13 in the serum-free conditioned medium from melanoma cells

Variant	A375[a]	B16-F10[a]
pcDNA3-Negative Control	13.267 ± 1.724	12.467 ± 1.986
pcDNA3-MMP-13	25.733 ± 2.954*	24.033 ± 1.436*
pRNAT-Negative Control	14.267 ± 1.380	12.400 ± 0.900
pRNAT-MMP-13 siRNA	7.633 ± 0.839**	6.533 ± 0.651**

[a]Concentration of active MMP-13 in the serum-free conditioned medium of A375 or B16-F10 melanoma cells (ng/ml)
*$P < 0.05$, compared with the cells transfected with pcDNA3-Negative Control ($n = 3$);
**$P < 0.05$, compared with the cells transfected with pRNAT-Negative Control ($n = 3$).
